# Personality traits and health-related quality of life: the mediator role of coping strategies and psychological distress

**DOI:** 10.1186/s12991-018-0196-0

**Published:** 2018-06-06

**Authors:** Angela J. Pereira-Morales, Ana Adan, Sandra Lopez-Leon, Diego A. Forero

**Affiliations:** 1 Laboratory of Neuropsychiatric Genetics, Biomedical Sciences Research Group, School of Medicine, Universidad Antonio Nariño, Bogotá, 110231 Colombia; 2 Department of Clinical Psychology and Psychobiology, School of Psychology, University of Barcelona, Barcelona, Spain; 3 Institute of Neurosciences, University of Barcelona, Barcelona, Spain; 4 Novartis Pharmaceuticals Corporation, One Health Plaza, East Hanover, NJ 07936-1080 USA

**Keywords:** Five-factor personality model, Coping, Health-related quality of life, Latin America, Mental health

## Abstract

**Background:**

The study of health-related quality of life (HRQOL) is an important topic in mental health around the globe. However, there is the need for more evidence about the cumulative influence of psychological variables on HRQOL. The main aim of the study was to evaluate how specific personality traits might explain scores in HRQOL and to explore how this relationship might be mediated by coping styles and psychological distress.

**Methods:**

Young Colombian subjects (*N* = 274) were included (mean age: 21.3; SD = 3.8). The Short-Form Health Survey was used to measure HRQOL. For assessment of psychological variables, the Hospital Anxiety and Depression Scale, the Zung Self-Rating Anxiety Scale, The Coping Inventory for Stressful Situations and the short version of Big Five Inventory were used.

**Results:**

The personality trait that was the best predictor of HRQOL was openness to experience, forming an explanatory model for HRQOL, along with emotional coping style and depressive and anxious symptoms. Emotional coping style and psychological distress were significant mediators of the relationship between openness and HRQOL.

**Conclusions:**

Our findings provide additional data about the cumulative influence of specific psychological variables on HRQOL, in a mostly young female Latin American sample.

**Electronic supplementary material:**

The online version of this article (10.1186/s12991-018-0196-0) contains supplementary material, which is available to authorized users.

## Background

Health-related quality of life (HRQOL) has been defined by the World Health Organization as: “An individual’s perception of their position in life, in the context of the culture and values in which they live and in relation to their goals, expectations, standards, and concerns’’ [1]. In developing countries, a low HRQOL has been associated with lower socioeconomic status (SES) [2]. Research has shown that a lower SES is associated with a poor mental health in young adults; this relationship can be explained by several risk factors, such as disadvantageous work characteristics, reduced social support and risky health behaviors [3–6].

In general, young people in South American countries are exposed to many vulnerabilities, such as inadequate access to education and social and health services, high rates of violence and a relatively easy access to drugs [7]. These susceptibilities might have important impacts on mental health [8], given the fact that psychosocial vulnerabilities during youth have been shown to have short- and long-term implications for the individuals and the society [8].

In terms of adaptation and coping, the emotional and cognitive evaluations of life satisfaction are important factors for understanding HRQOL [9]. Coping typically includes cognitive and behavioral strategies that are used to overcome or to resolve problematic life circumstances (e.g., problem solving) [10]. In addition, coping strategies are used to manage the emotional consequences of stressful situations [10]. In accordance with previous works [11], coping can be understood through three main dimensions or styles: task oriented, emotional, and avoidant, which represents self-reported responses to stressful circumstances. The task-oriented style is represented by subjects who generally take a problem-solving approach to stressful situations. In contrast, those who habitually engage in maladaptive behaviors, such as ruminating activities or becoming emotional in response to stress, have a predominantly emotional style. On the other hand, individuals who typically employ behaviors aimed at circumventing the stressful situation have a predominantly avoidant style [12]. Although there is evidence for stability in coping styles over time, they can change across the life span and across different stressful situations and the effect of the coping strategies depends on the specific situations [10, 13].

Personality is an important factor for the perception of stressful events and is considered as fundamental for having the required resources to cope in an unexpected situation [14]. Several studies have established evidence of associations between coping styles and personality [15], showing that the neurotic trait is positively correlated with the avoidant coping strategies and negatively correlated with the task-oriented coping style, while openness has been associated with active coping strategies, such as seeking social support [16–23].

The study of HRQOL is an important topic in mental health worldwide, taking into account its relationship with subjective well-being and other health outcomes [9]. Although HRQOL is influenced by life circumstances and demographic characteristics (such as socioeconomic status), more evidence is needed about the cumulative effect of multiple psychological variables on physical and mental health-related quality of life. The main aim of the current study was to test, in a sample of young Colombian subjects, the hypothesis that specific personality traits might explain scores in HRQOL and that this relationship might be mediated by coping styles and psychological distress.

## Materials and methods

### Participants

Two hundred seventy-four young Colombian healthy subjects were included in this study. Recruitment of the participants started with an invitation in two universities in Bogotá, Colombia. The aims and procedures of the study were explained to the interested subjects and they were invited for the application of the psychosocial evaluations. The mean of age was 21.3 years (standard deviation, SD = 3.8), 75.1% were females and 24.9% were males. The participants did not have a personal history of neuropsychiatric disorders, according to self-report. The study followed the ethical standards of the Helsinki Declaration and all subjects signed a written informed consent, with prior approval of the study by the Institutional Ethics Committee of the Antonio Nariño University.

### Psychological scales and instruments

HRQOL was assessed with the 12-Item Short-Form Health Survey (SF-12). The SF-12 is a self-report scale that provides a reliable measure of the perception of physical (PCS) and mental (MCS) health. PCS dimension includes the physical functioning, physical health problems, general health and bodily pain domains. Meanwhile, MCS dimension includes the social functioning, emotional problems, mental health and vitality domains [24]. The scores are standardized to population norms (based on a European normative sample), with the mean score set at 50 (SD = 10); scores above 50 indicate better perception of health status [25]. The method used to compute values for the two main dimensions (PCS and MCS) was based on the algorithm provided by Andersen et al. [26], which was based on the procedure described in the SF-12 manual. The SF-12 has been validated in Spanish and in Colombia, with adequate reliability and psychometric properties [27]. In the current study, the Cronbach’s alphas were 0.74 and 0.72 for PCS and MCS, respectively.

Psychological distress was assessed using the Hospital Anxiety and Depression Scale (HADS) and the Zung Self-Rating Anxiety Scale (ZSAS). We used two scales for the assessment of anxiety symptomatology since ZSAS includes somatic aspects of anxiety that HADS-A does not evaluate. In addition, HADS-A assesses generalized anxiety symptoms, such as tension, worry, panic, difficulties in relaxing, and restlessness. The *HADS* is a self-report screening instrument created to indicate the possible presence of anxiety and depression states. It includes two sub-factors: Depression (HADS-D) and Anxiety (HADS-A), each one with 7 items. It has demonstrated excellent reliability and validity in English and Spanish languages, including Colombia [28]. The cutoff points are ≥ 6 for depression, ≥ 8 for anxiety and ≥ 13 for the total test. The Cronbach’s alpha for the current study was 0.83 for the total HADS score, 0.77 for the anxiety subscale and 0.65 for the depression subscale. The ZSAS is an instrument that provides a self-report of symptoms, based on the characteristic signs of anxiety. It has shown excellent reliability and validity in Spanish and in Colombia [29]. The Cronbach’s α for the ZSAS was 0.85 in this sample.

Coping was measured with the Coping Inventory for Stressful Situations (CISS-SF), in the short form version with 21 items [30]. This inventory assesses three different dimensions or coping styles (task-oriented, emotional, and avoidant). CISS items exemplify different ways of coping in a particular stressful situation. Good internal consistency has been found for its subscales in the English language and it has been previously used in the Spanish language [30]. In the current study, the internal consistency of the emotional coping style was *α* = 0.84, of 0.80 for task-oriented style and of 0.65 for the avoidant style.

To assess personality dimensions, the Big Five Inventory (BFI-S; 15-items) was used [31]. The Big Five personality trait model is one of the most established and used approaches to measure individual differences in personality. This self-report inventory measures five dimensions of personality: N (Neuroticism), E (Extraversion), O (Openness to experience), C (Conscientiousness) and A (Agreeableness) on a 7-point Likert scale. It has been validated in the English language by Lang et al. [31] and it has been widely used in several countries, such as Spain [32]. In the current study, the Cronbach’s alpha was 0.61 for Extraversion, 0.73 for Openness, 0.42 for Consciousness, 0.47 for Agreeableness and 0.62 for Neuroticism. The instruments selected for this work (SF-12, HADS, ZSAS, CISS-SF and BFI-S) are reliable and efficient tools for psychosocial measurement, which are broadly used and have been validated in Spanish.

### Statistical analysis

Normal distributions of the scores for the used scales were explored with previously described methods, including analyses of skewness and kurtosis [33]. The psychometric properties of the instruments used were evaluated using Cronbach’s alpha and exploratory factorial analysis.

The association of the HRQOL (total SF-12 scores and PCS/MCS dimensions) and predictor variables (psychological distress, coping and personality traits) was examined using Pearson correlations and multiple regression models, controlling for age and gender. Collinearity was examined with the variance inflation factor (VIF) and independence assumption on the residuals was evaluated with the Durbin–Watson test. For a better characterization of HRQOL and its predictors, two models of multiple regression analyses were conducted: (1) one for SF-12/Physical Component Score and (2) one for SF-12/Mental Component Score.

The Statistical Package for the Social Sciences V. 18 (SPSS Inc., Chicago, Illinois, United States) was used for all the statistical analyses. The Bonferroni correction for multiple testing [34] was included. For the current study, the Bonferroni corrected *p* value for the regression models 1 and 2 was 0.012.

Mediation analysis was performed to evaluate the mediator role of coping styles and psychological distress in the relationship between HRQOL and personality traits. Only significant predictors for HRQOL in the regression model were included in mediation analysis. Following the procedures recommended by Hayes [35], mediation-in-serial models using multiple regressions with three mediators were carried out. Direct effects, indirect effects, and total effects were calculated using specification model 6 with three mediators in the PROCESS plugin (V.2014) [35] in SPSS (V.18).

Openness was inserted as an independent variable, HRQOL was included as an outcome variable and emotional coping style, task-oriented coping style and psychological distress (depressive and anxious symptoms) were inserted as the mediator variables. To assess the magnitude of the indirect effects, we report *partially standardized indirect effects* of the independent variable on the outcome variable. According to Preacher et al. [36], this index is interpreted as the number of standard deviations by which the outcome variable is expected to increase or decrease per each change in independent variable indirectly via the mediator variables.

## Results

### Descriptive data and Associations between variables

The majority of the participants were from low and medium SES (33.5 and 44.5%, respectively). The most common education level and marital status of the subjects were secondary (78.8%) and single (93%), respectively. Sixteen and thirteen percent of the subjects showed lower scores of PCS and MCS, respectively. The mean scores for the psychological distress scales were 13.9 (SD = 5.4) for HADS scale and 38.5 (SD = 9.4) for ZSAS. The preferred coping style was task oriented (mean = 22.7; SD = 5.0), followed by emotional (mean = 19.9; SD = 6.0) and avoidant (mean = 17.6; SD = 5.1). In terms of the personality traits, Factor O showed the highest mean value (5.4; SD = 1.0), followed by factor C (mean = 4.9; SD = 1.0). Neuroticism was the personality trait with the lowest average value (4.1; SD = 1.2).

Pearson’s correlations are summarized in Table S1. HRQOL/PCS was negatively correlated with task-oriented coping style, openness and conscientiousness (*r* = − 0.171, *r* = − 0.175, *r* = − 0.136, *p* < 0.01, respectively) and positively correlated with emotional coping style and neuroticism (*r* = 0.194, *r* = 0.169, *p* < 0.01). HRQOL/MCS was negatively correlated with psychological distress (measured with HADS and ZSAS) and with emotional coping style and neuroticism (*r* = − 0.568, *r* = − 0.547, *r* = − 0.533, *r* = − 0.442, *p* < 0.01, respectively) and correlated positively with task-oriented coping style, openness, conscientiousness, extraversion and agreeableness (*r* = 0.254, *r* = 0.205, *r* = 0.139, *r* = 0.211, *r* = 0.187, *p* < 0.01, respectively).

Task-oriented coping style was negatively correlated with psychological distress (measured with HADS and ZSAS), emotional coping style and neuroticism (*r* = − 0.303, *r* = − 0.328, *r* = − 0.133, *r* = − 0.228, *p* < 0.01, respectively) and it was positively correlated with openness, conscientiousness and agreeableness (*r* = 0.310, *r* = 0.291, 0.312, *p* < 0.01, respectively). Finally, emotional coping style positively correlated with psychological distress (measured with HADS and ZSAS) and with neuroticism (*r* = 0.509, 0.576, 0.486, *p* < 0.01, respectively) and avoidance coping style correlated with extraversion (*r* = 0.222, *p* < 0.01).

### Multivariate analyses

#### Model 1

The best regression model for HRQOL/PCS was composed by emotional coping style (*β *=0.28, *p* = 0.0001), openness (*β *=-0.14, *p* = 0.018), anxiety symptoms (measured with ZSAS, *β *=− 0.19, *p* = 0.008) and task-oriented coping style (*β *=− 0.15, *p* = 0.017) (Table 1).

**Table 1:** Stepwise multiple regression models for HRQOL and associated psychological factors

Variable	Model 1 HRQOL (SF-12 PCS)^a^			Model 2 HRQOL (SF-12 MCS)^b^		
	*β*	SE	*R* ^2^	*β*	SE	*R* ^*2*^
Emotional coping style	0.19**	7.1	0.03			
Emotional coping style × openness	0.18** × − 0.16**	7.0	0.05			
Emotional coping style × openness × ZSAS	0.27** × − 0.18** × − 0.14*	6.9	0.07			
Emotional coping style × Openness × ZSAS × Task-oriented coping style	0.28** × − 0.14* × − 0.19** × − 0.15*	6.9	0.08			
HADS				− 0.58**	14.0	0.32
HADS × emotional coping style				− 0.40** × − 0.33**	13.2	0.39
HADS × emotional coping style × ZSAS				− 0.30** × − 0.27** × − 0.18**	13.0	0.41
HADS × emotional coping style × ZSAS × openness				− 0.27** × − 0.28** × − 0.17** × 0.10*	12.9	0.42

*ZSAS* Zung Self-Rating Anxiety Scale, *HADS* Hospital Anxiety and Depression Scale, *SE* standard error

* Significant at *p* < 0.05

** Significant after Bonferroni correction for multiple testing

^a^Model 1: Outcome variable measured with 12-Item Short-Form Health Survey-Physical Component Score

^b^Model 2: Outcome variable measured with 12-Item Short-Form Health Survey-Mental Component Score

#### Model 2

For HRQOL/MCS, the best regression model found was composed by psychological distress (measured with HADS total score, *β *= − 0.27, *p* = 0.00003), ZSAS (*β *=− 0.17, *p* = 0.010), emotional coping style (*β *=− 0.28, *p* = 1.4508E^−06^) and openness (*β *=0.10, *p* = 0.025) (Table 2).

**Table 2:** Multiple mediation effect for HRQOL (SF-12 PCS) and HRQOL (SF-12 MCS)

		Coefficient	CI lower	CI upper	Effect size (95% CI)
Mediation model 1: HRQOL (SF-12 PCS)	Indirect effect via M1^a^	0.204	0.030	0.507	0.028 (0.003–0.068)
	Indirect effect via M1 and M2^b^	− 0.169	− 0.401	− 0.039	− 0.023 (− 0.054 to − 0.005)
	Indirect effect via M1 and M3^c^	− 0.051	− 0.159	− 0.004	− 0.007 (− 0.022 to − 0.0006)
	Indirect effect via M2 and M3^d^	− 0.003	− 0.039	0.003	− 0.0005 (− 0.005–0.0004)
	Indirect effect via M1, M2 and M3^e^	0.006	− 0.003	0.035	0.0008 (− 0.0005–0.0049)
	Total indirect effect	− 0.203	− 0.622	0.170	− 0.028 (− 0.084–0.023)
Mediation model 2: HRQOL (SF-12 MCS)	Indirect effect via M1	0.126	− 0.004	0.351	0.025 (0.0001–0.0696)
	Indirect effect via M1 and M2	0.406	0.082	0.944	0.023 (0.004–0.053)
	Indirect effect via M1 and M3	0.377	0.075	0.970	0.022 (0.004–0.052)
	Indirect effect via M2 and M3	− 0.041	− 0.206	0.270	− 0.002 (− 0.011–0.0016)
	Indirect effect via M1, M2 and M3	0.071	0.014	0.256	0.004 (0.0009–0.0136)
	Total indirect effect	1624	0.323	3.019	0.095 (0.017–0.175)

Model 1: M1 = mediator 1 (anxiety symptoms measure with ZSAS); M2: mediator 2 (emotional coping style); M3: mediator 3 (task-oriented coping style). Model 2: M1 = mediator 1 (anxiety symptoms measure with ZSAS); M2: mediator 2 (emotional coping style); M3: mediator 3 (psychological distress measure with HADS)

*CI* confidence interval

^a^The indirect effect of the openness on health-related quality of life via anxiety symptoms measure with ZSAS

^b^The indirect effect of the Openness on Health-related Quality of Life via anxiety symptoms measure with ZSAS and emotional coping style

^c^The indirect effect of the openness on health-related quality of life via anxiety symptoms measure with ZSAS and task-oriented coping style

^d^The indirect effect of the openness on health-related quality of life via emotional coping style and task-oriented coping style

^e^The indirect effect of the openness on health-related quality of life via anxiety symptoms measure with ZSAS, Emotional coping style and Task-oriented coping style

In this sample of young adults, anxiety symptoms, emotional coping style, task-oriented coping style and openness explained 8% of the variance on HRQOL in its physical dimension; while psychological distress (anxiety and depressive symptoms), emotional coping style and openness explained 42% of the variance on HRQOL in its mental dimension.

### Direct and indirect effects of openness on HRQOL Physical Component Score via coping styles and anxious symptoms

**Fig. 1:**
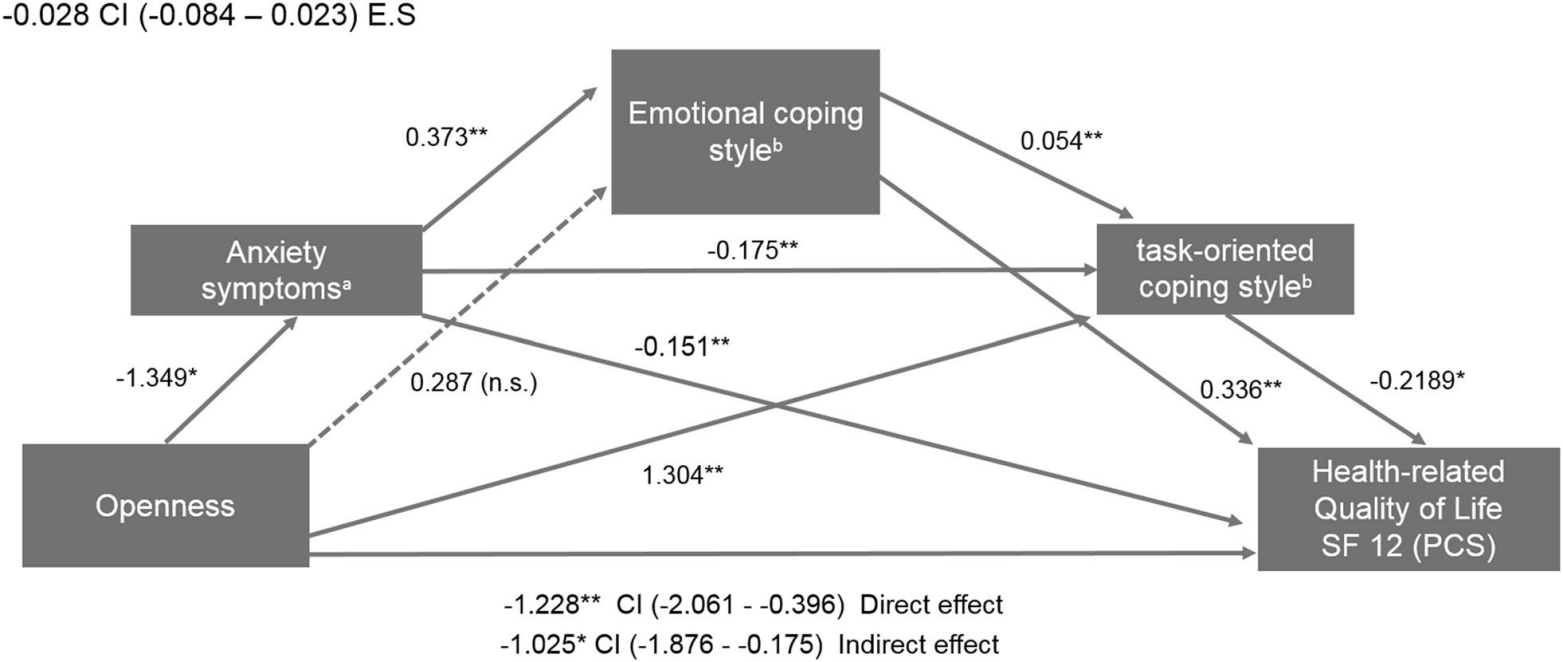
Path diagram of sequential mediation for HRQOL (SF-12 Physical Component Score). ^a^Anxiety symptoms measured with ZSAS (Zung Self-Rating Anxiety Scale). ^b^Coping styles measured with the Coping Inventory for Stressful Situations (CISS-SF). *E.S* effect size of total indirect effect for the mediation model, *CI* confidence interval, *n.s.* no significant. *p < 0.05; **p < 0.001

The first mediation model (Fig. 1) tested the relationship of openness to experience with HRQOL/PCS, via the effects of task-oriented coping style, emotional-focused coping, and ZSAS. Openness affected HRQOL/PCS directly (coefficient: − 1.228, SE = 0.42, *p* = 0.0040) (Fig. 1), as well as indirectly through emotional coping style, task-oriented coping style and anxiety symptoms measured with ZSAS (coefficient: − 1.0257, SE = 0.43, *p* = 0.0183). The total indirect effect of openness on HRQOL/PCS, via emotional coping style, task-oriented coping style, and anxiety symptoms was not significant (coefficient: − 0.203, 95% CI − 0.622 to 0.170) (Table 2).

### Direct and indirect effects of openness on HRQOL Mental Component Score via emotional coping style and psychological distress

**Fig. 2:**
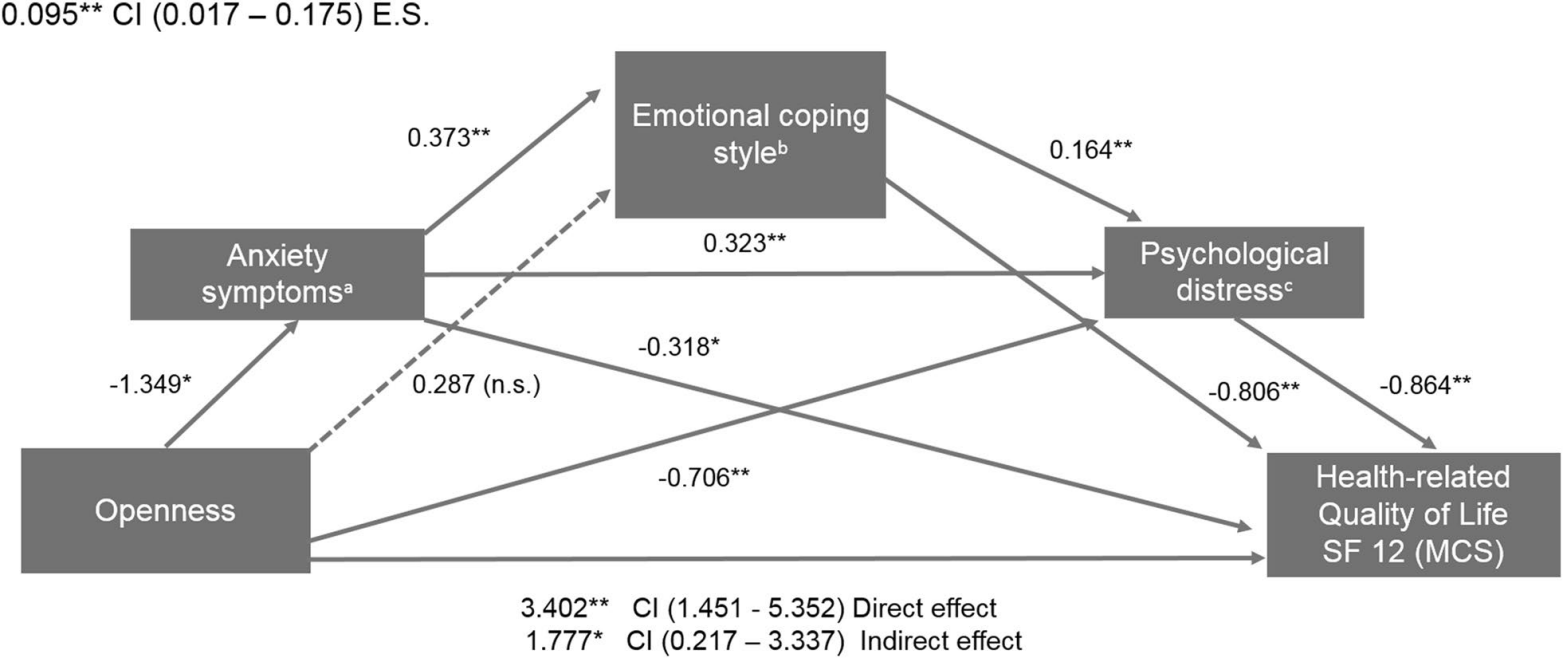
Path diagram of sequential mediation for HRQOL (SF-12 Mental Component Score). ^a^Anxiety symptoms measured with Zung Self-Rating Anxiety Scale (ZSAS). ^b^Coping styles measured with the Coping Inventory for Stressful Situations (CISS-SF). ^c^Psychological distress measured with psychological distress was assessed using the Hospital Anxiety and Depression Scale. *E.S* effect size of total indirect effect for the mediation model, *CI* confidence interval, *n.s.* no significant. *p < 0.05; **p < 0.001

The second mediation model (Fig. 2) tested the relationship of openness to experience with HRQOL/MCS, via emotional-focused coping, total HADS score, and ZSAS. Openness affected HRQOL (SF-12 MCS) directly (coefficient: 3.402, SE = 0.99, *p* = 0.0007), as well as indirectly via emotional coping style and psychological distress (coefficient: 1.777, SE = 0.79, *p* = 0.0257) (Table 2). The total indirect effect of openness on HRQOL/MCS through emotional coping style and psychological distress (measured with HADS and ZSAS) was significant (coefficient: 1.624, 95% CI 0.323–3.019) (Table 2).

The partially standardized indirect effect for openness on subjective well-being (SF-MCS) via emotional coping style and psychological distress (measured with ZSAS and HADS) was 0.095 (95% CI 0.017–0.175). It implies that HRQOL/MCS is expected to increase by 0.095 standard deviations for every increase in one unit in openness, indirectly through emotional coping style and psychological distress. Other significant effect sizes for the indirect effects were found (Table 2).

## Discussion

In this work, in a sample of young Colombian subjects, we tested the hypothesis that specific personality traits might explain scores in HRQOL and that this relationship might be mediated by coping styles and psychological distress. We found that the personality trait that best predicted HRQOL was openness, forming an explanatory model for HRQOL, along with emotional coping style and depressive and anxious symptoms. We also found that emotional coping style and psychological distress were significant mediators of the relationship between openness and HRQOL.

Over the past three decades, there has been considerable progress on research on health-related quality of life [37]. Previous studies have indicated that HRQOL is influenced by both personality and coping [38]. However, there is the need for inclusion of other relevant psychological variables, in addition to personality and coping, in the analytical models and studies [39]. This is important taking into account that HRQOL is a multidimensional construct that has been under-investigated for a long time in young populations in developing countries [9]. The results for HRQL in the current sample are similar to findings in other studies [23]. Our results provide a multidimensional model for HRQOL, which includes personality, coping and emotional variables and that explains a considerable percentage of HRQOL variance, in a Colombian sample of young adults.

The relationship between personality traits and HRQOL has been reported in previous studies, finding that personality is one of the strongest and most consistent predictors for HRQOL in the general population [40]. Specifically, it has been suggested that neuroticism has an interactive effect on HRQOL, as well that agreeableness, conscientiousness, and extraversion have been also predictors of self-rated health [41]. Openness to experience has the fewest documented links to health; nevertheless, recent studies have found higher levels of openness as protective against earlier mortality [42].

It has been proposed that personality may affect the selection of coping strategies directly or indirectly, facilitating the use of specific approaches [16]. Some investigations have found that certain personality traits are related to specific coping styles. Neurotic subjects (characterized by high reactivity to stress) generally show emotional or avoidant coping strategies [17]. On the other hand, individuals who have low-stress reactivity, such as subjects with high scores in consciousness and openness personality traits, have been shown to prefer task-oriented coping strategies [43].

Previous studies have shown a positive association between openness and approach coping strategies [15] and other reports found an association between approach coping strategies and increased distress and non-productive worry [15]. These previous results are consistent with our findings of psychological distress as a mediator of the relationship between openness and HRQOL. Moreover, both emotional coping style and psychological distress were explanatory factors (negatively correlated) for HRQOL (physical and mental dimensions). In addition, task-oriented coping style was a significant factor for the HRQOL physical dimension. Although the effect of the coping strategies depends on the specific situations, these patterns of relationships might indicate that individuals with high scores in openness can experience a recovery from the initial emotional reaction to a stressful event and move on to deal with the source of the stress more quickly and effectively than neurotic individuals do, who generally prefer emotional coping strategies and show low scores in HRQOL. This is supported in part by a previous study [44] and by another report that has shown that openness reflects a more flexible, imaginative, and intellectually curious approach to problem solving [45]. On the other hand, in our second model of mediation, when emotional coping style and with psychological distress were included, the relationship between openness and HRQOL mental dimension was diminished. This finding can be explained in terms of the evidence that has shown that passive coping strategies, such as emotional coping style, involve avoidance and withdrawal behaviors, instead of a rational approach in dealing with difficulties, which is more characteristic of subjects with higher scores in openness experience [46]. In turn, it is well known that healthy adaptive strategies are positively associated with active coping behaviors and with a better perception of quality of life [47]; and are inversely associated with maladaptive strategies, such as emotional-focused styles, and with lower levels of openness experience [48, 49].

Nonetheless, in some cases, emotional coping strategies may also be a protective factor. For example, some emotional coping style strategies, such as acceptance or religion, can help to reduce depressive symptoms and contribute to HRQOL [50], particularly if a problem is unlikely to be resolved. However, other avoidant coping attitudes, such as denial and substance use, are often less useful and have been associated with impulsivity and anxious symptoms [51].

A considerable amount of previous studies have documented the impact of neuroticism and conscientiousness on objective measures of health and its perception [52], but few previous studies have shown evidence that higher levels of openness are also a significant factor for perception of HRQOL. Future studies would help to confirm the role of personality and coping in HRQOL processes, which might help to keep people healthy as they move across the decades of adulthood [52].

In the context of coping strategies, personality traits are considered an important factor for having the required resources to cope in stressful situations [14] as well as in the perception of stressful events and on HRQOL. In our sample, openness, conscientiousness, and agreeableness were significantly correlated with the task-oriented coping style. In our study, openness, task-oriented coping style, anxiety symptoms and emotional coping style formed an associative model for HRQOL in its physical dimension. This is relevant because the task-oriented coping style is a strategy employed usually more frequent in subjects who generally take an active problem-solving approach to stressful situations [11]. A task-oriented coping style, in conjunction with conscientiousness, agreeableness and openness, may be an important protective factor for affective disorders [53].

In our mediation analyses, the relationship between HRQOL-MCS and openness (with emotional coping style and psychological distress as mediators) showed a significant effect size. This may suggest that these factors are more important variables for HRQOL-MCS, in comparison with HRQOL-PCS. Openness was significant (direct effect) in the mediation model for HRQOR-MCS after a Bonferroni correction for multiple testing. This is relevant because an openness to experience involves the tendency to be creative, curious, flexible, and inclined toward new activities and ideas [54]. These tendencies may facilitate engagement in coping strategies that require considering new perspectives, such as cognitive restructuring and problem solving [15].

This study has some limitations: the effect sizes found were small and these findings should be treated with caution, because mediation analysis is employed for testing casual relationships in experimental designs where is possible to conclude that causality has occurred. When using this method in behavioral studies, we should consider that the relationships in such contexts might be reciprocal, as it is possible that the independent variable affects the mediator and that the mediator might influence the independent variable as well [55]. In addition, the nature of our sample, composed mainly by females and educated participants, does not allow the generalization of the results to the general population (Additional file 1).

In future studies, it will be important to carry out an analysis of the genetic and epigenetic factors involved in HRQOL, as well as the possible interaction of these factors with environmental influences on HRQOL and perceived mental health.

## Conclusion

In this study, we tested the hypothesis that specific personality traits might explain scores in HRQOL and that this relationship might be mediated by coping styles and psychological distress. Our study provides novel mediational models that may help to understand the association between personality and coping in the context of HRQOL, in both their physical and mental dimensions. In addition, our findings provide additional data about the cumulative influence of specific psychological variables on health-related quality of life in a Latin American sample, composed mainly by young females.

## Additional file

**Additional file 1.** Pearson’s correlations for each component of HRQOL, distress, coping and personality variables.
