# Supplementary material for: Personality traits and health-related quality of life: the mediator role of coping strategies and psychological distress
Source: Ann Gen Psychiatry. 2018 Jun 6;17:25. doi: 10.1186/s12991-018-0196-0 (PMC5991445; doi:10.1186/s12991-018-0196-0)
Supplement: Supplementary file 1 — Additional file 1. Pearson’s correlations for each component of HRQOL, distress, coping and personality variables. [file 12991_2018_196_MOESM1_ESM.docx]

**Table S1.** Pearson's correlations for each component of HRQOL, distress, coping and personality variables.

|  | **HRQOL PCS** | **HRQOL MCS** | **Task-oriented coping style** | **Emotional coping style** | **Avoidance coping style** |
| --- | --- | --- | --- | --- | --- |
| *Task-oriented coping style* | -0.171** | 0.254** |  |  |  |
| *Emotional coping style* | 0.194** | -0.533** | -0.133** |  |  |
| *Openness* | -0.175** | 0.205** | 0.310** |  |  |
| *Conscientiousness* | -0.136** | 0.139** | 0.291** |  |  |
| *Neuroticism* | 0.169** | -0.442** | -0.228** | 0.486** |  |
| *Extraversion* |  | 0.211** |  |  | 0.222** |
| *Agreeableness* |  | 0.187** | 0.312** |  |  |
| *HADS* |  | -0.568** | -0.303** | 0.509** |  |
| *ZSAS* |  | -0.547** | -0.328** | 0.576** |  |

Note: **p<0.01; HRQOL: Health related quality of life; PCS: Physical component score; MCS: Mental component score.
